# Umbilical cord blood androgen levels and ASD-related phenotypes at 12 and 36 months in an enriched risk cohort study

**DOI:** 10.1186/s13229-017-0118-z

**Published:** 2017-01-31

**Authors:** Bo Y. Park, Brian K. Lee, Igor Burstyn, Loni P. Tabb, Jeff A. Keelan, Andrew J. O. Whitehouse, Lisa A. Croen, Margaret D. Fallin, Irva Hertz-Picciotto, Owen Montgomery, Craig J. Newschaffer

**Affiliations:** 1 Department of Mental Health, Johns Hopkins Bloomberg School of Public Health, 624 N Broadway HH884, Baltimore, MD 21205 USA; 2 Department of Epidemiology and Biostatistics, Drexel University School of Public Health, 3215 Market Street, Philadelphia, PA 19104 USA; 3 Department of Environmental and Occupational Health, Drexel University School of Public Health, 3215 Market Street, Philadelphia, PA 19104 USA; 4 School of Women’s and Infants’ Health, University of Western Australia, 35 Stirling Hwy, Crawley, WA 6009 Australia; 5 Telethon Kids Institute, University of Western Australia, 100 Roberts Rd, Subiaco, WA 6009 Australia; 6 Kaiser Permanente Division of Research, 2000 Broadway, Oakland, CA 94612 USA; 7 The MIND (Medical Investigations of Neurodevelopmental Disorders) Institute, University of California Davis, One Shields Ave. Med-Sci 1C, Davis, CA 95616 USA; 8 Department of Obstetrics and Gynecology, Drexel University College of Medicine, 219 N. Broad St, Philadelphia, PA 19107 USA; 9 A.J. Drexel Autism Institute, 3020 Market St. Suite 560, Philadelphia, PA 19104 USA

**Keywords:** Autism, Testosterone, Sex difference, Umbilical cord blood, Sibling

## Abstract

**Background:**

Autism spectrum disorder (ASD) affects more than 1% of children in the USA. The male-to-female prevalence ratio of roughly 4:1 in ASD is a well-recognized but poorly understood phenomenon. An explicit focus on potential etiologic pathways consistent with this sex difference, such as those involving prenatal androgen exposure, may help elucidate causes of ASD. Furthermore, the multi-threshold liability model suggests that the genetic mechanisms in females with ASD may be distinct and may modulate ASD risk in families with female ASD in the pedigree.

**Methods:**

We examined umbilical cord blood from 137 children in the Early Autism Risk Longitudinal Investigation (EARLI) cohort. EARLI is an ASD-enriched risk cohort with all children having an older sibling already diagnosed with ASD. Fetal testosterone (T), androstenedione (A4), and dehyroepiandrosterone (DHEA) levels were measured in cord blood using liquid chromatography-tandem mass spectrometry (LC-MS/MS). Robust linear regression models were used to determine associations between cord blood androgen levels and 12-month Autism Observation Scales for Infants (AOSI) scores and 36-month Social Responsiveness Scale (SRS) scores adjusting for potential confounders.

**Results:**

Increasing androgens were not associated with increasing 12-month AOSI score or 36-month total SRS score in either boys or girls. However, the association between T and autistic traits among subjects with a female older affected sibling was greater at 12 months (test of interaction, *P* = 0.008) and deficits in reciprocal social behavior at 36 months were also greater (test of interaction, *P* = 0.006) than in subjects whose older affected sibling was male.

**Conclusions:**

While increased prenatal testosterone levels were not associated with autistic traits at 12 or 36 months, our findings of a positive association in infants whose older ASD-affected siblings were female suggests an androgen-related mechanism that may be dependent on, or related to, genetic liability factors present more often in families containing female ASD cases. However, this initial finding, based on a small subgroup of our sample, should be interpreted with considerable caution.

**Electronic supplementary material:**

The online version of this article (doi:10.1186/s13229-017-0118-z) contains supplementary material, which is available to authorized users.

## Background

The CDC estimates that 1 in 68 8-year-old US children have an autism spectrum disorder (ASD) [1]. The observed male-to-female prevalence ratio of approximately 4:1 in the CDC data is consistent with the vast majority of other epidemiologic studies around the world [2]. Two of the hallmark characteristics of ASD, impaired social interaction and communication, have been recognized as traits that show sex differences in typical development [3, 4], although debate continues around the exact magnitude, nature, and generalizability of these differences [5]. While this striking ASD sex difference has long been acknowledged, the mechanisms underlying this difference remain largely unknown.

The nature of sex differences in cognition and behavior among typically developing children has led to the Extreme Male Brain theory of autism, which proposed that ASD is an extreme presentation of a typical male cognitive profile where the drive to “systemize” is stronger than the drive to empathize. A mechanism involving sex steroid exposure could be consistent with the Extreme Male Brain theory [6]. Evidences from animal research indicate that altering prenatal sex-typical steroid levels can lead to permanent changes in the developing brain [7]. Androgens, especially testosterone, are produced at higher levels in the male fetus compared to female fetus, and androgens have an established role in fetal brain development and are associated with sex-related differences in behavior [3, 7, 8]. The predominant source of fetal testosterone exposure is endogenous production by the fetal-placental unit [9] and, under normal circumstances, only small amounts of maternal testosterone passively diffuse across the placenta [10, 11]. In typically developing male fetuses, prenatal testosterone levels are higher than in female fetuses, particularly between 8 and 24 weeks of gestation. There is also a neonatal testosterone surge after birth reaching pubertal levels during first 3 months, which then declines to pre-pubertal levels by 6 months of age [12].

Girls with congenital adrenal hyperplasia (CAH) are exposed to elevated androgen concentrations and have been studied as a model of elevated prenatal androgen exposure in females. Girls with CAH show a more male-typical profile compared to unaffected females in gender-typed play across multiple studies [5, 13–15]; however, findings regarding differences in cognitive abilities such as mental spatial rotation [16–18] and language learning [18, 19] have been less consistent.

The multiple-threshold liability model explains the sex difference in ASD prevalence as a result of females having a higher genetic threshold for attaining ASD case status than males and, therefore, female cases carry a higher mutational load [20]. Two studies of high-risk children with ASD siblings did not observe differences in ASD recurrence by the older ASD sibling’s sex [21, 22]. However, a large population-based dizygotic twin pair study observed that siblings of autistic females show greater autistic impairments than siblings of autistic males [23], and a similar increase in average symptom severity on the Social Responsiveness Scale among ASD cases in families with female cases was observed in a high-risk study of children with ASD siblings [24].

Researchers attempting to measure fetal testosterone have assayed amniotic fluid and umbilical cord blood. Amniotic fluid surrounding the fetus is collected via amniocentesis, which is generally performed between 15 and 20 weeks of gestation when the prenatal testosterone levels substantially differ across sexes. Amniocentesis samples are limited to pregnancies with indications for this procedure, however, which creates potential selection bias if the indications for amniocentesis are associated with outcomes under study. Moreover, the exact relationship between steroid levels in amniotic fluid and those in fetal circulation are not known. Umbilical cord blood is an easily collected biological sample reflecting late-term prenatal testosterone, available from any pregnancy. Testosterone levels measured in cord blood are thought to reflect prenatal levels in late gestation after the peak in males [25].

Several previous ASD studies have used amniotic fluid in an attempt to determine fetal testosterone levels. Two studies found positive associations between amniotic fluid testosterone levels and increased autistic traits among 18–24 month olds and 4 year olds [26, 27]; however, this was not replicated in a recent study among 3 to 5 year olds [28]. Another recent birth cohort-based case control study of males found no difference in amniotic fluid testosterone between cases and controls but observed increased amniotic fluid steroidogenic activity, measured as principal components of cortisol and steroids levels along the testosterone synthesis pathway, across cases and controls [29]. Two published population-based studies of cord blood testosterone level and ASD-related traits from one group found no association [30, 31]. However, these studies were limited by assessment of the phenotype later in life (~20 years), and thus subjects were exposed to decades of postnatal environmental and developmental influences on behavior, which could influence the ability to detect an association.

The current study aims to investigate the association between umbilical cord blood levels of testosterone and other related androgens and autistic traits measured at 12 months of age and social impairment assessed at 36 months of age in the Early Autism Risk Longitudinal Investigation (EARLI), an enriched ASD risk pregnancy cohort. In addition, we aim to explore whether this association was modified by the sex of the older affected sibling.

## Methods

### Description of study sample

The Early Autism Risk Longitudinal Investigation (EARLI) is a high autism-risk cohort following pregnant mothers with an older child diagnosed with an ASD (autistic disorder, Asperger syndrome, or pervasive developmental disorder not otherwise specified). EARLI families were recruited at four EARLI Network sites (Drexel/Children’s Hospital of Philadelphia; Johns Hopkins/Kennedy Krieger Institute; UC Davis; and Northern California Kaiser Permanente) in three distinct US regions (Southeast Pennsylvania, Northeast Maryland, and Northern California). In addition to having a biological child with an ASD confirmed by EARLI study clinicians, eligible mothers also had to communicate in English or Spanish and, at recruitment, meet the following criteria: be 18 years or older; live within 2 h of a study site; and be less than 29 weeks pregnant. The design of the EARLI study is described in more detail in Newschaffer et al. [32]. Mothers were provided with sampling kits for cord blood collection prior to delivery. EARLI research staff made arrangements with obstetricians/midwives and birth hospital labor and delivery staff to assure proper sample collection and temporary storage. The development of children born into the cohort was closely followed. For this study, 212 infants born into EARLI and followed to 1 year of age were considered for inclusion. Outcomes were autistic traits assessed at 12 and 36 months measured by the Autism Observation Scale for Infants (AOSI) [33] and the Social Responsiveness Scale (SRS) [34], respectively. The study protocol also included general neurodevelopmental measures at these time points not analyzed here (e.g., the Mullen Scales of Early Learning and Vineland Adaptive Behavior Scales). Of the 212 infants, 75 were excluded from principal analyses because they were missing either umbilical cord blood samples or one of the two principal outcome measures, leaving a final sample of 137 infants. We also repeated key analyses on all available subjects with each individual outcome who also had cord blood available (175 subjects had AOSI-umbilical cord sample and 140 subjects with SRS-umbilical cord sample). Demographic characteristics were similar for those included and excluded from the study.

### Laboratory methods

Testosterone (T), androstenedione (A4), and dehydroepiandrosterone (DHEA) and internal standards ^2^H_3_-testosterone (^2^H_3_-T) and 19-^2^H_3_-androstenedione (^2^H_3_-A4) were extracted from cord blood using liquid-liquid extraction. T, A4, DHEA, ^2^H_3_-T, and ^2^H_3_-A4 were measured using a liquid chromatography-tandem mass spectrometry (LC-MS/MS) assay and a calibration curve linear across the range of the samples as previously described (CPR Pharma Services Pty Ltd, Thebarton, SA) [35]. Limit of quantification (LOQ) was specified as a minimum signal-to-noise ratio of at least 5:1, with quality controls at the LOQ successfully quantified within 20% of its nominal concentration. The LOQ values were 0.17, 0.35, and 3.47 nmol/L, respectively, for T, A4, and DHEA. Distributions of T, A4, and DHEA were all positively skewed, and log_e_-transformed values were used for the analysis. Geometric mean and geometric standard deviation were used to describe and compare the log_e_-transformed androgen levels. Study characteristics were compared across infant sex using Wilcoxon rank-sum tests for non-normally distributed continuous variables and *t* tests for normally distributed continuous variables. A *χ* ^2^ test was used to test for differences between categorical variables.

### Outcome assessment

ASD-related phenotype at 12 months was assessed using the AOSI, a semi-structured direct observational measure that takes approximately 20 min to complete [34, 36]. There are 16 items in the AOSI that are observed in the domains of visual tracking and attention disengagement, coordination of eye gaze and action, imitation, affective responses, early social-communicative behaviors, behavioral reactivity, and sensory-motor development [36]. These items are rated on three to four point scales and summed to generate a total score ranging from 0 to 19, with higher scores indicating more autistic traits. Total AOSI score has predicted subsequent ASD diagnosis in other high-risk sibling cohort studies [37, 38]. EARLI study clinicians engaged in regular exercises to maintain cross-site reliability in AOSI administration.

ASD-related phenotype at 36 months was measured using the SRS pre-school version. The SRS is a caregiver-reported 65-item questionnaire on social awareness, social cognition, social communication, social motivation, and autistic mannerism administered at approximately 36 months of age [39]. The SRS total raw score range from 0 to 180 and has been used internationally in numerous studies of quantitative autism traits. The SRS is a well-established quantitative measure of traits and symptoms in both the general population [40] and in ASD siblings [41] that is able to distinguish ASD children from both non-affected children and those with other conditions, such as mental retardation, with high internal validity, reliability, and reproducibility using the established SRS score thresholds [34, 42]. It has been validated against the “gold standard” for diagnosis, the Autism Diagnostic Interview-Revised (ADI-R) [34, 43]. Use of the SRS in both general population and affected samples has demonstrated that SRS scores are continuously distributed and are not related to intelligence quotient or age [34, 39, 44, 45].

### Covariates

Covariates examined included maternal characteristics (age, education, race, ethnicity, total number of previous pregnancies), cesarean delivery, gestational age at delivery, and sex of the older affected sibling. Potential confounders of a priori interest were maternal age, gestational age, and cesarean delivery, all of which have been associated with both prenatal androgen level and ASD risk [35, 46–48]. Bivariate analyses were conducted between covariates and both outcomes as well as between covariates and exposure. Candidate covariates were not included in final adjusted models if they were not associated with both the outcome and exposure at a significance level of 0.1. Since we hypothesized that other biological differences between males and females could modify the association between testosterone levels and autistic traits, infant sex was examined as a potential effect modifier. Further, since genomic analyses suggest that multiplex families with affected females in the pedigree may have different genetic liability mechanisms than families with only male cases [23], the older sibling’s sex was also explored as a potential effect modifier.

### Statistical analyses

Separate robust linear regression models [49] were used to estimate the associations between each androgen level and the two principal outcome measures. Total AOSI scores were positively skewed, and log_e_ (ln)-transformed (AOSI + 1) values were used to account for subjects with zero AOSI score (*n* = 10, 7%). Similarly, total SRS scores were positively skewed and log_e_ (ln) transformation was also used. In all analyses, androgen values below the LOQ were replaced with estimates of the expected value of levels below LOQ (LOQ/$\sqrt{2}$: T = 0.12, A4 = 0.25, DHEA = 2.45). We estimated associations between ln(quantitative outcome) and ln(hormone concentration) in both unadjusted models and in sex-stratified models controlling for maternal age and gestational age. In models exploring effect modification by older sibling sex, infant sex was also included as a covariate. We also explored likelihood-based estimation (in R) and multiple imputation by chained equations (in SAS) approaches for handling LOQ, but in all instances, results were consistent with those based on simple replacement.

All analyses, except those noted above, were performed using STATA 12 [50]. This study was approved by the Drexel University Institutional Review Board (IRB) and informed consent was obtained from all participants and/or their parent/guardian in accordance with the Drexel University IRB approved protocol.

## Results

### Androgens and autism phenotype in boys versus girls

Overall and sex-stratified characteristics of the infant study sample (75 males, 62 females) are shown in Table 1. There were no observed differences in maternal age, education, race, ethnicity, total number of previous pregnancies, proportion of births from cesarean sections, or in the sex distribution of the older ASD-affected sibling by infant sex. Among the 137 cord blood samples analyzed, testosterone (T), androstenedione (A4), and dehyroepiandrosterone (DHEA) measured below the limits of quantification (LOQ) in 11, 1 and 14% of the samples, respectively (Table 2). Female infants had a higher proportion of T measuring below LOQ, and males had a higher proportion of DHEA measuring below LOQ (Table 2). Median values for T, A4, and DHEA were 0.44, 1.74, and 6.93 nmol/L, respectively (Table 2). All three androgens (T, A4, and DHEA) were positively correlated with each other, with the highest observed Pearson correlations in females being 0.69 between ln-T and ln-DHEA and the highest observed correlation in males being 0.60 between ln-A4 and ln-DHEA (Additional file 1: Figure S1). Levels of T were on average higher (*P* < 0.0001) in males (median = 0.61 nmol/L; interquartile range (IQR) = 0.40–0.79 nmol/L) versus females (median = 0.33 nmol/L; IQR = 0.21–0.48 nmol/L) (Table 2). Levels of A4 were similar in males and females (*P* = 0.23). Levels of DHEA were on average higher (*P* < 0.001) in females (median = 8.75 nmol/L; IQR = 5.99–11.58 nmol/L) compared to males (median = 5.85 nmol/L; IQR = 4.12–9.64 nmol/L) (Table 2).

**Table 1:** Study characteristics by infant sex

	Total (*n* = 137)		Female (*n* = 62)		Male (*n* = 75)		
Characteristics	Mean	SD	Mean	SD	Mean	SD	*P*
Maternal age	34.1	4.5	33.7	4.2	34.5	4.7	0.30
Gestational age at delivery	39.4	1.6	39.4	1.4	39.3	1.7	0.68
Total number of pregnancies	3.6	1.5	3.5	1.4	3.7	1.5	0.65
Total AOSI score^a^	5.0	4.3	4.7	2.0	5.2	2.3	0.45
Total SRS raw score^a^	29.8		26.1	1.7	33.4	2.1	0.03
	%	*n*	%	*n*	%	*n*	
Cesarean section	42.2	97	45.2	42	40	55	0.61
Maternal education		137		62		75	0.68
<9th	2.9		4.8		1.3		
High school graduation	8.0		8.1		8.0		
College degree	63.5		62.9		64.0		
Graduate/professional degree	25.6		24.2		26.7		
Maternal race		131		61		70	0.58
Asian	6.8		6.6		7.1		
Black	9.2		11.5		7.1		
Other	11.5		13.1		10.1		
White	64.9		59.0		70		
Unknown	7.6		9.8		5.7		
Maternal Hispanic/Latino	19.4	129	15.2	59	22.9	70	0.27
Male older affected sibling	83.9	137	85.5	62	82.7	75	0.66

^a^Geometric mean and geometric standard deviation

**Table 2:** Testosterone (T), androstenedione (A4), and dehydroepiandrostenedione (DHEA) levels (nmol/L) in umbilical cord samples

Infant sex^a^	Androgens	% <LOQ	Median	IQR
Total (*n* = 137)	T	11	0.44	0.12–0.64
	A4	1	1.74	1.42–2.27
	DHEA	14	6.93	4.72–10.89
Female (*n* = 62)	T	19	0.33	0.21–0.48
	A4	2	1.76	1.32–2.27
	DHEA	8	8.75	5.99–11.58
Male (*n* = 75)	T	4	0.61	0.40–0.79
	A4	1	1.74	1.48–2.33
	DHEA	19	5.85	4.12–9.64

T: *P* < 0.0001, A4: *P* = 0.23, and DHEA: *P* < 0.001

*LOQ* limit of quantification, *IQR* interquartile range

^a^Test of sex difference of geometric mean using *t* test

**Fig. 1:**
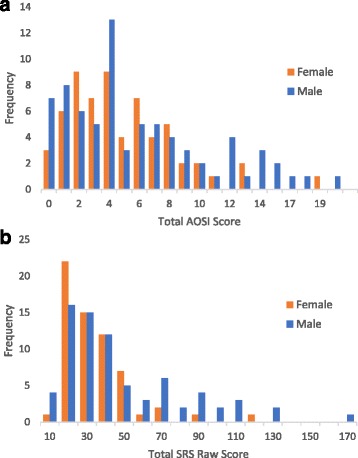
Distribution of 12- and 36-month outcomes by sex. **a** Twelve-month Autism Observation Scales for Infants (AOSI) total score (*n* = 137, female = 62, male = 75). **b** Thirty-six-month Social Responsiveness Scale (SRS) total raw score (*n* = 137, female = 62, male = 75)

Male infants had similar (*P* = 0.45) geometric mean AOSI score at 12 months (geometric mean (GM) = 5.2, geometric standard deviation (GSD) = 2.3) compared to females (GM = 4.7; GSD = 2.0), whereas the geometric mean 36-month SRS score was higher (*P* = 0.03) in males (GM = 33.4, GSD = 2.1) than in females (GM = 26.1, GSD = 1.7) (Table 1 and Fig. 1). Transformed 12-month AOSI and 36-month SRS were weakly correlated with Pearson’s *r* of 0.20, with observed correlations greater among males (*r* = 0.32) than females (*r* = 0.01).

**Fig. 2:**
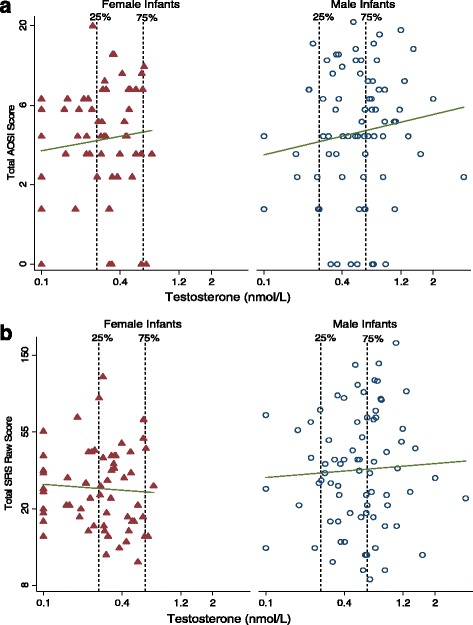
Relationship between umbilical cord testosterone level and 12- and 36-month outcomes by sex. **a** Twelve-month Autism Observation Scales for Infants (AOSI) total score; line fit to unadjusted log-log model; infant male (*circle*) and female (*triangle*) (*n* = 137, female = 62, male = 75). **b** Thirty-six-month Social Responsiveness Scale (SRS) total raw score; line fit to unadjusted log-log model; infant male (*circle*) and female (*triangle*) (*n* = 137, female = 62, male = 75)

Pearson correlations of ln-transformed androgens (T, A4, and DHEA) and AOSI were weak (*r* = 0.14, 0.04, and 0.03, respectively) and remained weak within sex (males: *r* = 0.13, 0.05, and 0.02, respectively; females: *r* = 0.11, −0.01, and 0.1, respectively). Pearson correlations of ln-transformed androgens (T, A4, and DHEA) and SRS score were also weak (*r* = 0.10, −0.12, and −0.08, respectively) and remained weak within sex (males: *r* = 0.05, −0.15, and −0.01, respectively; females: *r* = −0.06, −0.15, and −0.07, respectively). Figure 2 shows a sex-stratified scatterplot of cord blood T levels against AOSI and SRS scores. Scatterplots for A4 and DHEA are included in Additional file 1: Figures S2–S5.

### Associations of androgen levels and autism phenotype

Unadjusted robust linear regression models did not indicate any statistically significant associations between androgen levels and autism-related phenotype at either 12 or 36 months in infants of either sex (Table 3), and adjustment for maternal and gestational age did not alter these findings substantively (Table 4).

**Table 3:** Unadjusted regression models of androgen levels and 12- and 36-month outcomes within each sex

Androgens^a^	Infant sex	Outcomes					
		12-month AOSI			36-month SRS		
		Beta	95% CI	*P*	Beta	95% CI	*P*
ln(T)	Female (*n* = 62)	0.20	(−0.12,0.51)	0.22	−0.06	(−0.30,0.19)	0.64
	Male (*n* = 75)	0.20	(−0.13,0.52)	0.23	0.05	(−0.25,0.36)	0.73
ln(A4)	Female (*n* = 62)	0.06	(−0.36,0.48)	0.78	−0.18	(−0.50,0.14)	0.26
	Male (*n* = 75)	0.06	(−0.33,0.45)	0.76	−0.21	(−0.57,0.15)	0.24
ln(DHEA)	Female (*n* = 62)	0.09	(−0.19,0.37)	0.51	−0.05	(−0.25,0.16)	0.67
	Male (*n* = 75)	0.04	(−0.28,0.34)	0.82	−0.01	(−0.31,0.29)	0.94

*ln* natural log transformed

^a^Separate robust regression models of log_e_-transformed testosterone (T), androstenedione (A4), and dehydroepiandrostenedione (DHEA) with total log_e_ (AOSI + 1) and log_e_ (SRS raw) scores. Outcome measures are 12-month Autism Observation Scales for Infants (AOSI) total score and 36-month Social Responsiveness Scale (SRS) total raw score

**Table 4:** Total and infant sex-stratified adjusted models of androgen levels and 12- and 36-month outcomes

Androgens^a^	Subject sex	Outcomes							
		12-month AOSI				36-month SRS			
		Beta	95% CI	*P*	*P* ^b^	Beta	95% CI	*P*	*P* ^b^
ln(T)	Female (*n* = 62)	0.20	(−0.13,0.53)	0.22	0.95	−0.05	(−0.31,0.20)	0.67	0.85
	Male (*n* = 75)	0.22	(−0.13,0.57)	0.22		0.14	(−0.19,0.46)	0.41	
ln(A4)	Female (*n* = 62)	0.11	(−0.35,0.57)	0.64	0.90	−0.19	(−0.54,0.15)	0.27	0.89
	Male (*n* = 75)	0.05	(−0.37,0.47)	0.81		−0.17	(−0.55,0.21)	0.38	
ln(DHEA)	Female (*n* = 62)	0.1	(−0.18,0.39)	0.48	0.74	−0.04	(−0.26,0.17)	0.70	0.84
	Male (*n* = 75)	0.02	(−0.31,0.36)	0.88		−0.01	(−0.31,0.32)	0.97	

*ln* natural log transformed

^a^Robust regression models of log_e_-transformed testosterone (T), androstenedione (A4), and dehydroepiandrostenedione (DHEA) with total log_e_ (AOSI + 1) and log_e_ (SRS raw) adjusted for gestational age and maternal age. Outcome measures are 12-month Autism Observation Scales for Infants (AOSI) total score and 36-month Social Responsiveness Scale (SRS) total raw score

^b^Interaction *P* value comes from a model including both subject sexes including covariates, hormone variable, and subject sex hormone interaction

**Fig. 3:**
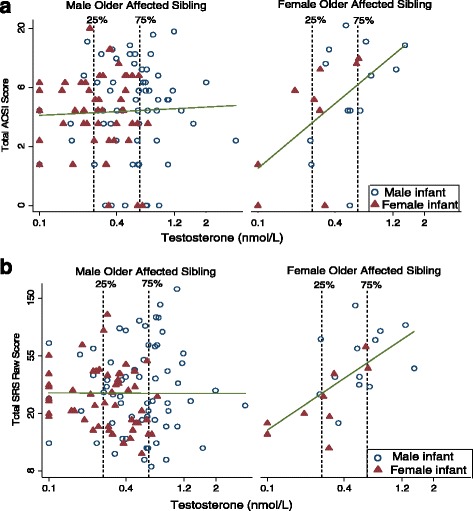
Scatterplot of testosterone level and 12- and 36-month outcomes by sex of the older ASD-affected child. **a** 12-month Autism Observation Scales for Infants (AOSI) total score; line fit to unadjusted log-log model (*n* = 137, female older affected sibling = 22, male older affected sibling = 115). **b** Thirty-six-month Social Responsiveness Scale (SRS) total raw score; line fit to unadjusted log-log model (*n* = 137, female older affected sibling = 22, male older affected sibling = 115)

Robust regression models stratifying by sex of the older ASD-affected sibling and adjusting for maternal and gestational age as well as infant sex, suggested that the association between T and ASD-related phenotype at both 12 and 36 months was stronger in those with a female affected older sibling, and non-stratified models incorporating interaction terms indicated that the interaction effects are statistically significant (Table 5). The stratified log-log models predict that in children with a female ASD-affected older sibling, a 25% increase in T is associated with a 23% increase in 12-month AOSI score (1.25^0.91^ = 1.23), while in children with a male ASD-affected older sibling the same increase in T is associated with just a 2% increase in 12-month AOSI score (1.25^0.08^ = 1.02). For 36-month SRS, this same 25% increase in T among children with a female ASD-affected older sibling is associated with a 15% increase in SRS score (1.25^0.61^ = 1.15), while in children with a male ASD-affected older sibling, a 2% decrease in SRS score (1.25^−0.08^ = 0.98) is predicted. For A4 and DHEA, the patterns of associations across subgroups defined by older affected sibling sex were similar, but the interactions were not statistically significant. Figure 3 shows the older ASD-affected sibling sex-stratified scatterplots of cord blood T levels against 12-month AOSI and 36-month SRS scores (scatterplots for A4 and DHEA are included as Additional file 1: Figures S6–S9). Additional file 1 includes results, presented in a comparable format to Tables 4 and 5, of models adding those subjects with only one available outcome measure (Additional file 1: Table S1). Results were similar in magnitude and direction to those in Tables 4 and 5, and the interaction between cord blood testosterone level and older affected sibling sex remained statistically significant for both outcomes (Additional file 1: Tables S2 and S3).

**Table 5:** Adjusted models of androgen levels and 12- and 36-month outcomes stratified by the older affected sibling’s sex

Androgens^a^	Older affected sibling’s sex	Outcomes							
		12-month AOSI				36-month SRS			
		Beta	95% CI	*P*	*P* ^b^	Beta	95% CI	*P*	*P* ^b^
ln(T)	Female (*n* = 22)	0.91	(0.20,1.63)	0.02	0.008	0.61	(0.27,0.94)	0.001	0.006
	Male (*n* = 115)	0.08	(−0.16, 0.32)	0.50		−0.08	(−0.30,0.14)	0.47	
ln(A4)	Female (*n* = 22)	0.22	(−1.88,2.33)	0.82	0.84	1.10	(−0.07,2.28)	0.06	0.06
	Male (*n* = 115)	0.05	(−0.22,0.33)	0.70		−0.23	(−0.48,0.02)	0.07	
ln(DHEA)	Female (*n* = 22)	0.39	(−0.40,1.18)	0.32	0.36	0.18	(−0.26,0.61)	0.40	0.68
	Male (*n* = 115)	0.02	(−0.20,0.24)	0.86		−0.06	(−0.26,0.14)	0.57	

*ln* natural log transformed

^a^Robust regression models of log_e_-transformed testosterone (T), androstenedione (A4), and dehydroepiandrostenedione (DHEA) with total log_e_ (AOSI + 1) and log_e_ (SRS raw) adjusted for infant sex, gestational age and maternal age. Outcome measures are 12-month Autism Observation Scales for Infants (AOSI) total score and 36-month Social Responsiveness Scale (SRS) total raw score

^b^Interaction *P* value comes from a model including both subject sexes including covariates, hormone variable, subject sex, older affected sibling sex and older affected sibling sex hormone interaction

## Discussion

We investigated the association between prenatal androgen exposure and autism-related quantitative phenotypes among infant siblings of older children previously diagnosed with ASD. Androgens were not associated with autistic traits at 12 months of age within each sex. Male infants showed significantly higher umbilical cord testosterone levels and greater social deficits at 36 months of age; however, elevated umbilical cord testosterone level was not associated with social impairments after adjusting for confounders in the full sample or in infants of either sex. These findings are consistent with previous reports on early adulthood autistic traits and cord blood testosterone level and testosterone to estrogen ratio in the general population [30, 31] but differed from some studies on early childhood autistic traits and testosterone in mid-gestation amniotic fluid samples [26, 27], which was not replicated in a recent study [51]. Our findings also suggest that the association between androgen levels and autistic traits may depend on sex of the older affected sibling with a positive association observed among infants with an older female affected sibling, after adjustment for infant sex as well as gestational and maternal age. This pattern of heterogeneity of effect by sex of the older affected sibling observed at 12 months persisted in analyses with 36-month outcomes.

While these findings of effect modification by older sibling sex must be considered as preliminary and need to be replicated, they are consistent with some prior findings suggesting that distinct etiologic mechanisms may be involved in multiplex families with female affected children. The empirical investigations published to date in multiplex ASD families have examined the influence of older affected sibling sex on recurrence risk, and two recent analysis reported higher recurrence rates in the next-born child when at least one of the previously affected children was female [24, 52]. This is consistent with an earlier report on two large samples of siblings of ASD-affected children that found higher levels of quantitative ASD phenotype in younger siblings of female probands than in younger siblings of male probands [23]. However, two large population-based studies [22, 53] and two smaller prospective studies of infant siblings of ASD-affected children [21, 54] found no differences in recurrence rates by older sibling sex.

Assuming multi-threshold liability, the model most commonly hypothesized to explain the sex difference in ASD prevalence, females have a higher genetic threshold for attaining ASD case status than males and, therefore, female cases carry a higher mutational load [20]. If some of these mutations are also related to androgen pathways, then these androgen-dependent mechanisms might be more commonly involved in families with affected females. To date, there have been more than ten genes associated with both autism and autism-related phenotype that are also associated with some aspect of sex steroid function (i.e., synthesis [55], transport [55], metabolism [55, 56], or feedback mechanism [57]). In addition, genetic variants might also modulate the susceptibility of the developing brain to endogenous testosterone. For example, there are two known polymorphisms in the androgen receptor gene (AR), located on the X chromosome [58], and, in one of these, the repeat sequence length has been inversely associated with receptor transcription activity [59, 60]. Shorter variants in this polymorphism have been linked to higher in vitro androgen receptor transcription activity in a kidney fibroblast cell line [59] and were more prevalent in ASD female cases compared to controls [60]. A recent study also indicated that some of the genes involved in naturally occurring sexually dimorphic processes are upregulated in ASD postmortem brains [61].

In further considering the results here, there are also a number of other issues and limitations. First, although we analyzed levels of three androgens separately, given the correlation between androgens and what is known about steroidogenesis, the specificity of individual hormone effects is not certain. Further, the current study relied on cord blood rather than amniotic fluid samples to evaluate androgen levels. These samples reflect late gestation androgen levels and do not capture exposure during the window where testosterone is higher in male fetuses than female fetuses, particularly from weeks 8 to 24 of gestation. Amniocentesis samples, however, are collected around the time of the large male-to-female prenatal testosterone level difference. If this sex difference in testosterone level was critical to brain changes influencing ASD-related behavior, then umbilical cord measurement of androgens would be weaker in testing hypotheses related to prenatal androgen exposure and ASD and could have contributed to our null result. While it has been assumed that the prenatal testosterone level difference is critical to testosterone’s influence on brain development, studies in nonhuman primates [62] show that levels of fetal testosterone later in pregnancy still independently influence sex-associated behaviors, and research in sheep [63] has identified late pregnancy windows where fetal testosterone influences structural and organizational changes in the brain [64]. Consequently, there may be multiple periods when the developing brain is susceptible to the influence of testosterone [64, 65].

Another potential limitation of the use of cord blood samples to measure androgens is that this sample commonly includes a mix of arterial and venous blood. While androgens in the umbilical artery primarily reflect circulating fetal androgens from fetal adrenal and gonad, those in the umbilical vein also reflect maternal and placental androgens [66]. However, a recent study comparing umbilical artery versus umbilical vein androgen levels found that while there are absolute differences between them, they are highly correlated (*r* = 0.67 ~ 0.83) [67]. Further, a recent population-based study of children of mothers with polycystic ovarian syndrome, which has hyperandrogenism as a defining characteristic, showed an elevated risk of autism in the child, suggesting that maternal prenatal androgen may also play a role in ASD risk [68].

In addition to androgen measurement issues, outcome measurement also deserves further reflection. Our findings were based on quantitative measures of ASD-related phenotype at 12 and 36 months, not categorical diagnostic classifications. However, twin studies have shown that the estimated magnitudes of genetic and non-shared environmental etiologic influences are quite similar regardless of whether outcome is a dichotomous or continuous ASD-related phenotype measure [69, 70]. This supports the idea that continuous and dichotomous ASD outcome measures are caused by common mechanisms.

We also must be mindful that the sample size here was modest, limiting the precision of effect estimates. In addition, the sample included only 22 subjects who had female older affected siblings and, therefore, the interesting results pertaining to this group, though statistically significant, are quite imprecise and this imprecision increases the plausibility that these results could be a chance finding. Lastly, as with most observational analyses, we cannot rule out the possibility that our findings may be influenced by residual confounding despite adjusting for known factors associated with exposure and outcome.

## Conclusions

We found that increased umbilical cord blood testosterone concentrations were not associated with autism-related phenotype at 12 or 36 months of age in a cohort of siblings of children with ASD. However, the existence of a positive association in the infants whose older affected siblings were female suggests an androgen-related mechanism that may be dependent on, or related to, genetic liability factors present more often in families containing female ASD cases. The number of subjects in our sample with older female affected siblings was small, and there was no a priori evidence supporting this particular sex-dependent mechanism, so these initial results should be interpreted cautiously.

## Additional file

Additional file 1: Figures S1–S5: Figure S1. Scatterplot between ln-transformed testosterone (T), androstenedione (A4), and dehydroepiandrostenedione (DHEA) levels by infant sex. **Figure S2.** Scatterplot between ln-transformed umbilical cord androstenedione level and total AOSI score by infant sex. **Figure S3.** Scatterplot between ln-transformed DHEA level and total AOSI score by infant sex. **Figure S4.** Scatterplot between ln-transformed androstenedione level and total SRS raw score by infant sex. **Figure S5.** Scatterplot between ln-transformed DHEA level and total SRS raw score by infant sex. **Figure S6.** Scatterplot between ln-transformed androstenedione (A4) level and total AOSI score by the older affected sibling’s sex. **Figure S7.** Scatterplot between ln-transformed androstenedione (A4) level and total SRS score by the older affected sibling’s sex. **Figure S8.** Scatterplot between ln-transformed DHEA level and total AOSI score by the older affected sibling’s sex. **Figure S9.** Scatterplot between ln-transformed DHEA level and total SRS raw score by the older affected sibling’s sex. **Tables S1–S3: Table S1.** Study characteristics comparison across two outcome measures. **Table S2.** Total and infant sex-stratified adjusted models of androgen levels with 12- and 36-month outcomes. **Table S3.** Adjusted models of androgen levels with 12- and 36-month outcomes stratified by the older affected sibling’s sex. (PDF 430 kb)

## Abbreviations

A4: Androstenedione
AOSI: Autism Observation Scales for Infants
ASD: Autism spectrum disorder
DHEA: Dehyroepiandrosterone
EARLI: Early Autism Risk Longitudinal Investigation
GM: Geometric mean
GSD: Geometric standard deviation
SRS: Social Responsiveness Scale
T: Testosterone
