# Supplementary material for: Umbilical cord blood androgen levels and ASD-related phenotypes at 12 and 36 months in an enriched risk cohort study
Source: Mol Autism. 2017 Jan 31;8:3. doi: 10.1186/s13229-017-0118-z (PMC5282802; doi:10.1186/s13229-017-0118-z)
Supplement: Additional file 1: Figures S1–S5: Figure S1. — Scatterplot between ln-transformed testosterone (T), androstenedione (A4), and dehydroepiandrostenedione (DHEA) levels by infant sex. Figure S2. Scatterplot between ln-transformed umbilical cord androstenedione level and total AOSI score by infant sex. Figure S3. Scatterplot between ln-transformed DHEA level and total AOSI score by infant sex. Figure S4. Scatterplot between ln-transformed androstenedione level and total SRS raw score by infant sex. Figure S5. Scatterplot between ln-transformed DHEA level and total SRS raw score by infant sex. Figure S6. Scatterplot between ln-transformed androstenedione (A4) level and total AOSI score by the older affected sibling’s sex. Figure S7. Scatterplot between ln-transformed androstenedione (A4) level and total SRS score by the older affected sibling’s sex. Figure S8. Scatterplot between ln-transformed DHEA level and total AOSI score by the older affected sibling’s sex. Figure S9. Scatterplot between ln-transformed DHEA level and total SRS raw score by the older affected sibling’s sex. Tables S1–S3: Table S1. Study characteristics comparison across two outcome measures. Table S2. Total and infant sex-stratified adjusted models of androgen levels with 12- and 36-month outcomes. Table S3. Adjusted models of androgen levels with 12- and 36-month outcomes stratified by the older affected sibling’s sex. (PDF 430 kb) [file 13229_2017_118_MOESM1_ESM.pdf]

Figure S1. Scatterplot between ln transformed umbilical cord testosterone (T), androstenedione (A4) and dehydroepiandrostenedione (DHEA) level by infant sex.

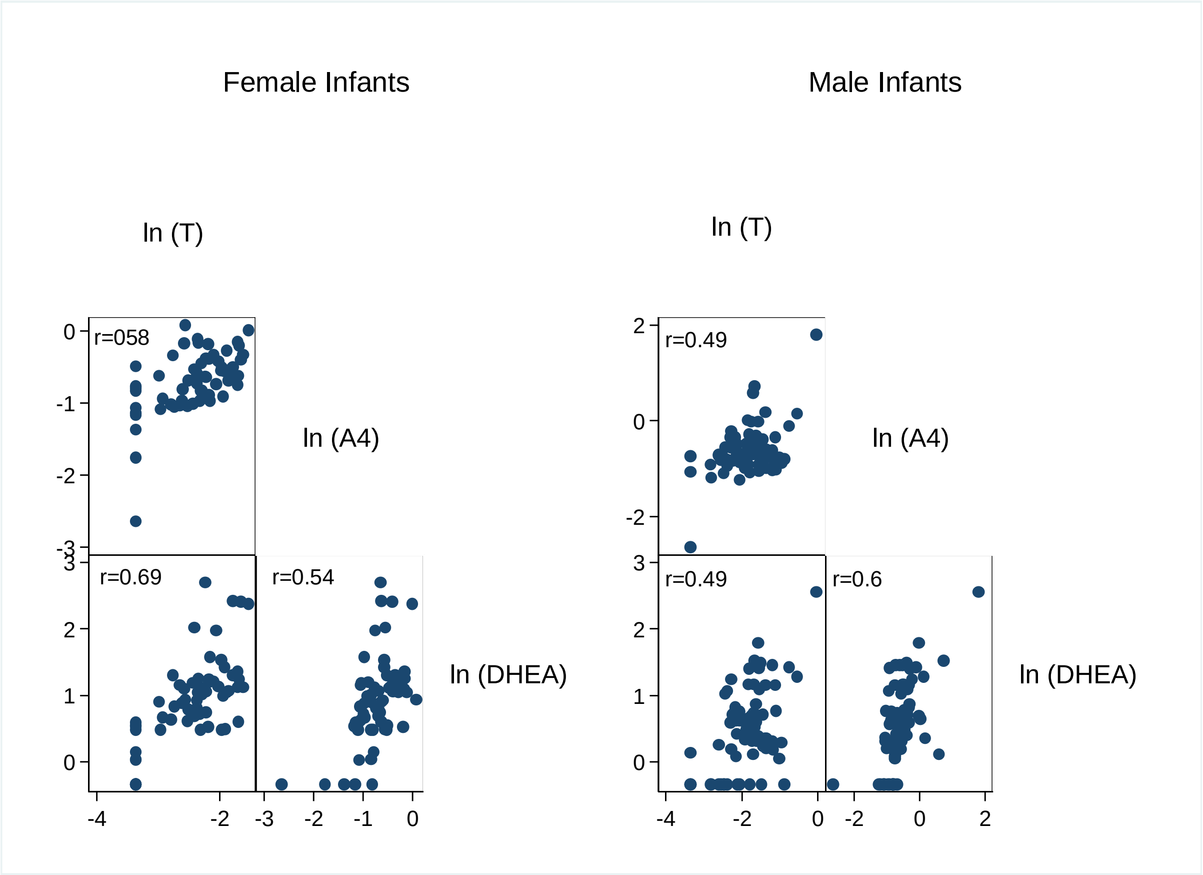

ln= natural log transformed; n=137 with Pearson's r, all with P value <0.001

Figure S2. Scatterplot between ln transformed umbilical cord androstenedione level and total AOSI score by infant sex.

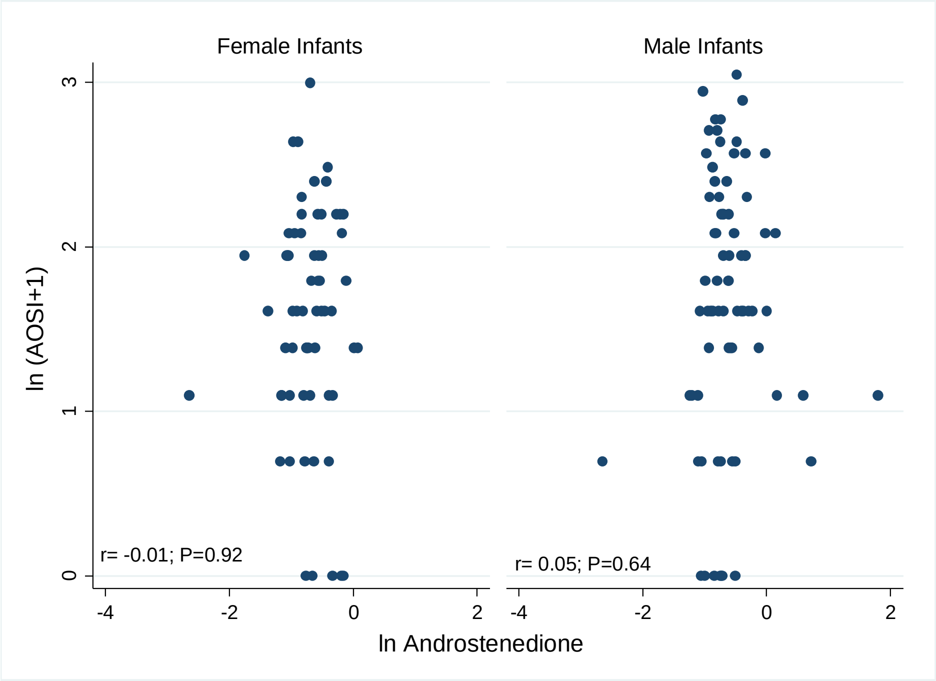

ln= natural log transformed; n=137 with Pearson's r and P value

Figure S3. Scatterplot between ln transformed umbilical cord dehydroepiandrostenedione (DHEA) level and total AOSI score by infant sex.

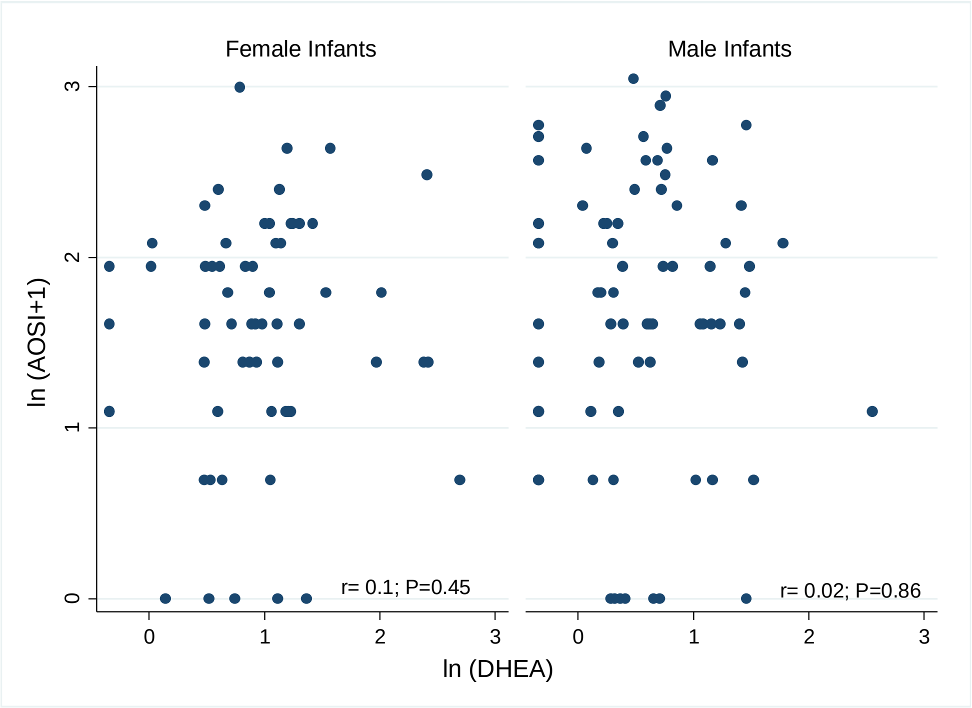

ln= natural log transformed; n=137 with Pearson's r and P value

Figure S4. Scatterplot between ln transformed umbilical cord androstenedione level and total SRS raw score by infant sex.

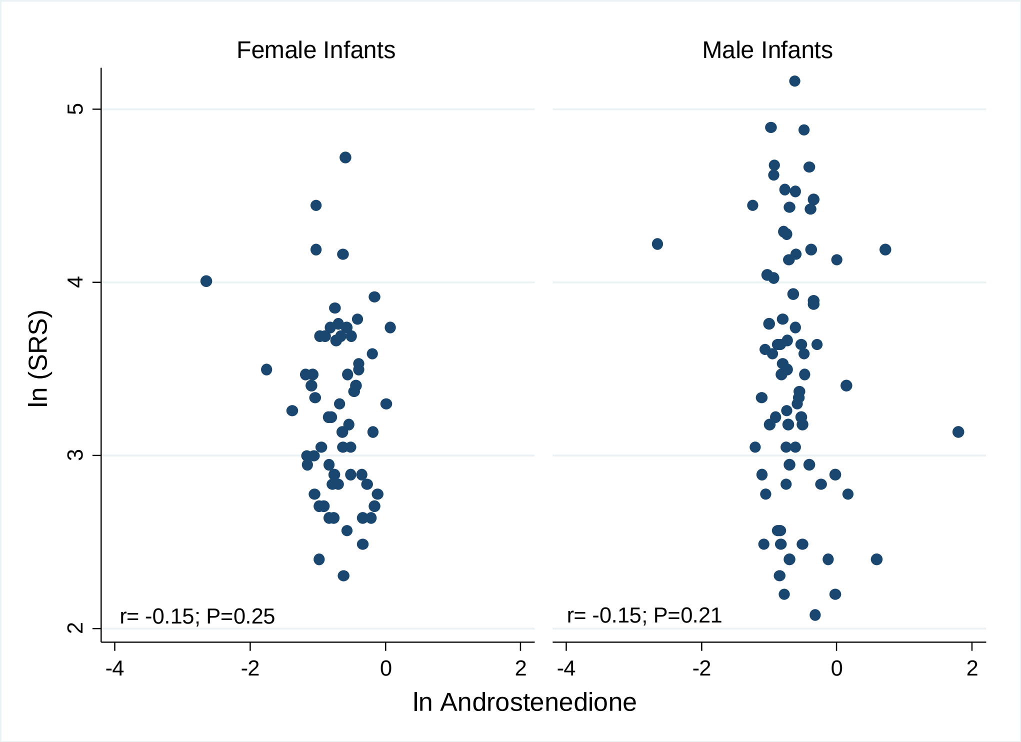

ln= natural log transformed; n=137 with Pearson's r and P value

Figure S5. Scatterplot between ln transformed umbilical cord dehydroepiandrostenedione (DHEA) level and total SRS raw score by infant sex.

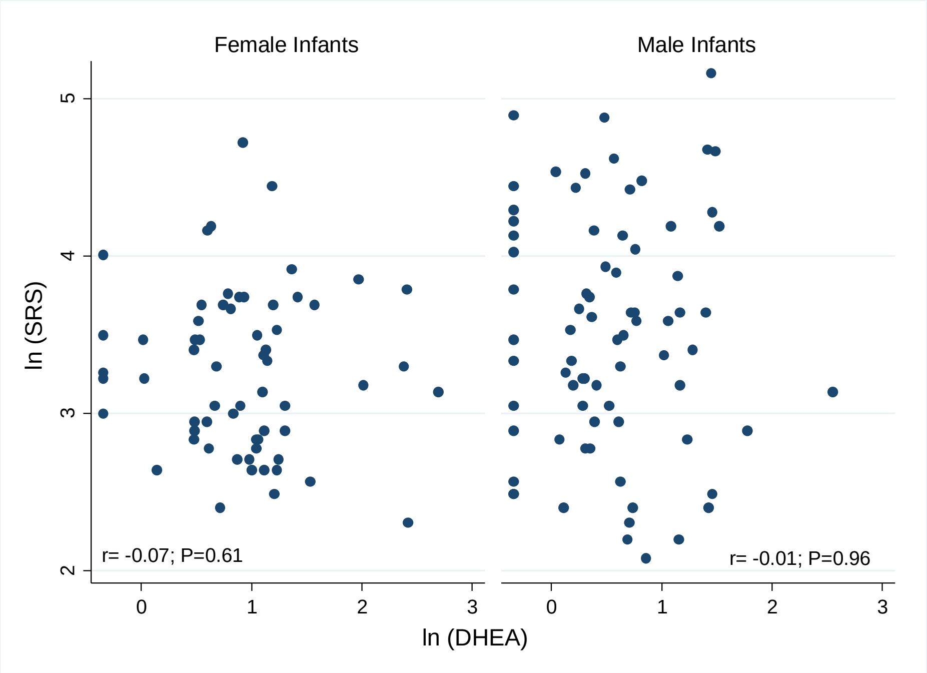

ln= natural log transformed; n=137 with Pearson's r and P value

Figure S6. Scatterplot between ln-transformed umbilical cord androstenedione (A4) level and total AOSI score by the older affected sibling's sex.

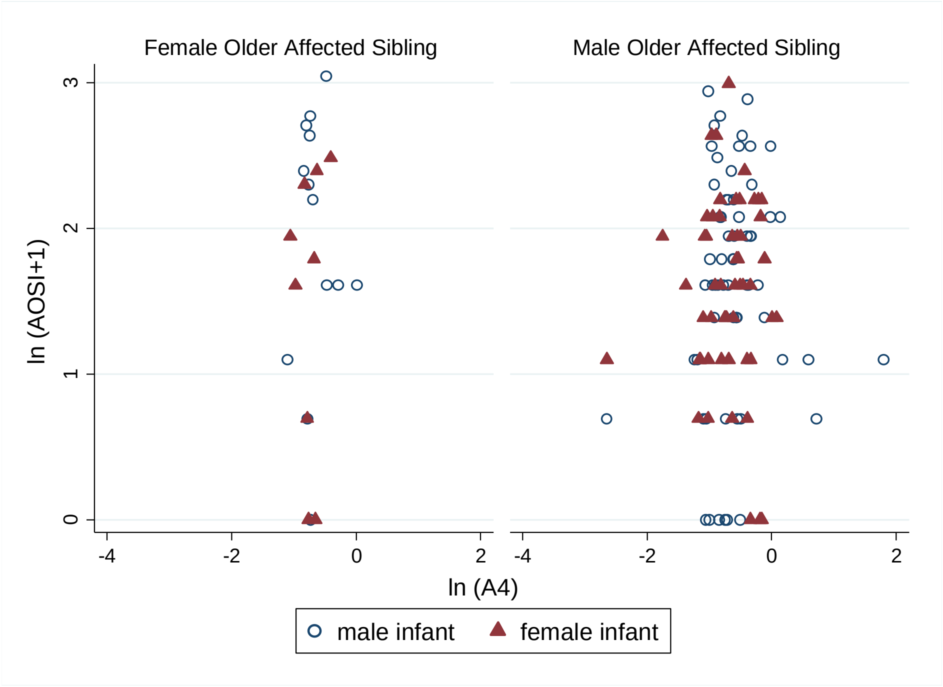

ln= natural log transformed; n=137, female older affected sibling=22, male older affected sibling=115

Figure S7. Scatterplot between ln-transformed umbilical cord androstenedione (A4) level and total SRS score by the older affected sibling's sex.

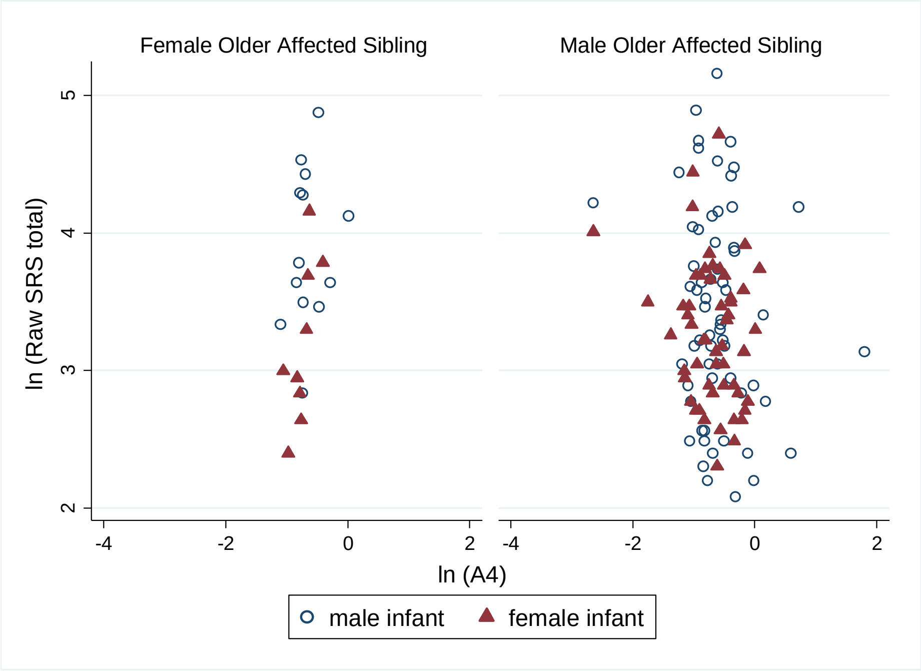

ln= natural log transformed; n=137, female older affected sibling=22, male older affected sibling=115

Figure S8. Scatterplot between ln-transformed umbilical cord dehydroepiandrostenedione (DHEA) level and total AOSI score by the older affected sibling's sex.

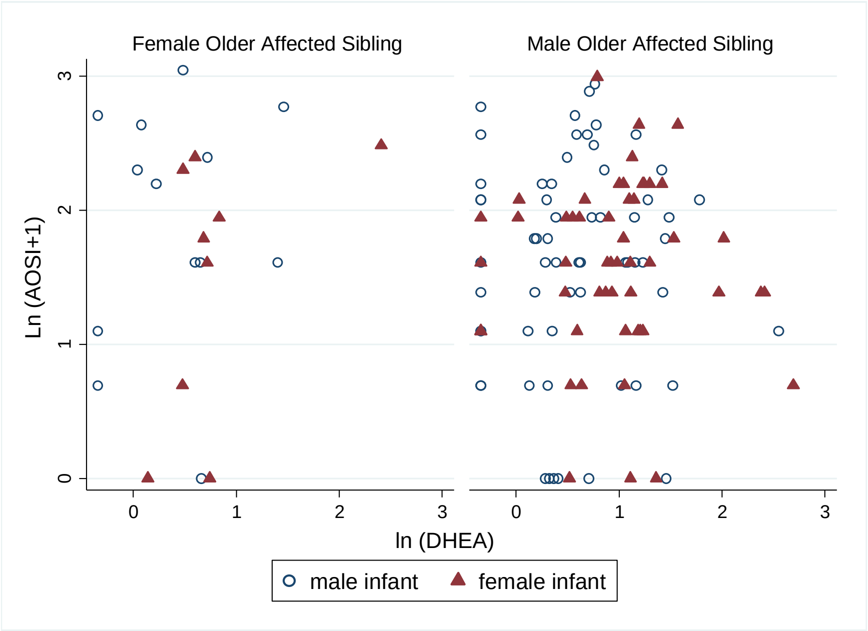

ln= natural log transformed; n=137, female older affected sibling=22, male older affected sibling=115

Figure S9. Scatterplot between ln-transformed umbilical cord dehydroepiandrostenedione (DHEA) level and total SRS raw score by the older affected sibling's sex.

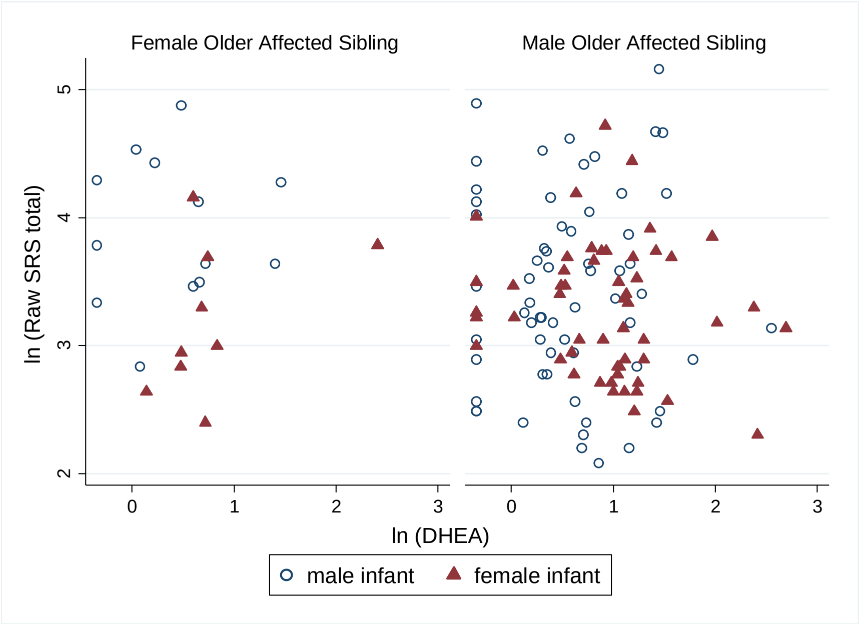

ln= natural log transformed; n=137, female older affected sibling=22, male older affected sibling=115

Table S1. Study characteristics comparison across two outcome measures.

Characteristics	AOSI only (n=175)		SRS only (n=140)		P
	Mean	SD	Mean	SD	
Maternal age	33.8	4.5	34.7	4.7	0.08
Gestational age at delivery	39.4	1.6	39.2	1.9	0.31
Total AOSI score*	5.26	4.3			
Total SRS Raw score*			28.8	1.96	
ln (Testosterone)*	0.43	1.94	0.42	1.93	0.94
ln (Androstenedione)*	1.81	1.59	1.80	1.62	0.96
ln (DHEA)*	6.93	1.98	7.10	1.95	0.45
	%	n	%	n	
Maternal Race		175		140	
Asian	11.2		10.8		0.96
Black	9.5		8.6		
Other	8.3		8.3		
White	64.5		64.2		
Unknown	5.9		7.9		
Maternal Hispanic/Latino	17.5	168	19.3	138	0.27
Older male affected sibling	85.1	175	83.6	140	0.72
Male infants	54.3	175	53.6	140	0.94

* Geometric mean and geometric sd is presented.
ln= natural log transformed.

Table S2. Total and infant sex stratified adjusted models of androgen levels with 12- and 36-month outcomes.

Androgens*	Subject Sex	Outcomes							
		12 month AOSI (Female=80, Male=95)				36 month SRS (Female=65, Male=75)			
		beta	95% CI	*P*	Interaction *P***	beta	95% CI	*P*	Interaction *P***
ln(T)	Female	0.20	(-0.10,0.50)	0.19	0.99	-0.06	(-0.30,0.18)	0.64	0.36
	Male	0.21	(-0.08,0.51)	0.16		0.14	(-0.19,0.46)	0.41	
ln(A4)	Female	0.13	(-0.31,0.56)	0.55	0.81	-0.20	(-0.53,0.13)	0.23	0.87
	Male	0.15	(-0.20,0.50)	0.38		-0.17	(-0.55,0.20)	0.36	
ln(DHEA)	Female	0.1	(-0.17,0.37)	0.47	0.77	-0.04	(-0.24,0.16)	0.71	0.83
	Male	0.07	(-0.18,0.31)	0.58		-0.01	(-0.31,0.31)	0.98	

*Robust regression models of $\log_e$ transformed testosterone (T), androstenedione (A4), and dehydroepiandrostenedione (DHEA) with total $\log_e$ (AOSI+1) and $\log_e$ (SRS raw) adjusted for infant sex, gestational age and maternal age. Outcome measures are 12-month Autism Observation Scales in Infants (AOSI) total score and 36-month Social Responsiveness Scale (SRS) total raw score.
**Interaction P value comes from a model including both subject sexes including covariates, hormone variable, subject sex, older affected sibling sex and older affected sibling sex*hormone interaction.
ln= natural log transformed

Table S3. Adjusted models of androgen levels with 12- and 36-month outcomes stratified by the older affected sibling's sex.

Androgens*	Older Affected Sibling's Sex	Outcomes							
		12 month AOSI (Female older affected sibling=26, Male older affected sibling =149)				36 month SRS (Female older affected sibling=23, Male older affected sibling=117)			
		beta	95% CI	*P*	Interaction *P***	beta	95% CI	*P*	Interaction *P***
ln(T)	Female	0.54	(-0.17,1.26)	0.13	0.02	0.57	(0.24,0.89)	0.002	0.004
	Male	0.10	(-0.11, 0.32)	0.34		-0.08	(-0.29,0.13)	0.33	
ln(A4)	Female	-0.03	(-1.65,1.59)	0.97	0.63	1.11	(-0.04,2.26)	0.06	0.06
	Male	0.10	(-0.15,0.34)	0.41		-0.23	(-0.48,0.02)	0.07	
ln(DHEA)	Female	0.10	(-0.44,0.65)	0.69	0.63	0.17	(-0.23,0.57)	0.39	0.84
	Male	0.06	(-0.13,0.25)	0.53		-0.06	(-0.26,0.14)	0.57	

*Robust regression models of $\log_e$ transformed testosterone (T), androstenedione (A4), and dehydroepiandrostenedione (DHEA) with total $\log_e$ (AOSI+1) and $\log_e$ (SRS raw) adjusted for infant sex, gestational age and maternal age. Outcome measures are 12-month Autism Observation Scales in Infants (AOSI) total score and 36-month Social Responsiveness Scale (SRS) total raw score.

**Interaction P value comes from a model including both subject sexes including covariates, hormone variable, subject sex, older affected sibling sex and older affected sibling sex*hormone interaction.

ln= natural log transformed
